# Effects of Dynamic Sitting Exercise with Delayed Visual Feedback in the Early Post-Stroke Phase: A Pilot Double-Blinded Randomized Controlled Trial

**DOI:** 10.3390/brainsci12050670

**Published:** 2022-05-20

**Authors:** Masahide Inoue, Kazu Amimoto, Kohei Shida, Daisuke Sekine, Daichi Hasegawa, Kazuhiro Fukata, Yuji Fujino, Shigeru Makita, Hidetoshi Takahashi

**Affiliations:** 1 Department of Rehabilitation, Saitama Medical University International Medical Center, 1397-1 Yamane, Hidaka, Saitama 350-1298, Japan; masahide.750@gmail.com (M.I.); tsurunan18@yahoo.co.jp (K.S.); s.daisuke1012@gmail.com (D.S.); dh97911@5931.saitama-med.ac.jp (D.H.); fkazuhiro.68@gmail.com (K.F.); smakita@saitama-med.ac.jp (S.M.); takataka1959@nifty.com (H.T.); 2 Department of Physical Therapy, Tokyo Metropolitan University, 7-2-10 Higashiogu, Arakawa-ku, Tokyo 116-8551, Japan; 3 Department of Physical Therapy, Juntendo University, 2-1-1 Hongo, Bunkyo-ku, Tokyo 113-8421, Japan; y.fujino.pb@juntendo.ac.jp

**Keywords:** stroke, sitting position, postural balance, paresis, feedback

## Abstract

Sitting ability in the early post-stroke phase affects functional balance ability and other prognoses. We investigated whether dynamic sitting exercise with delayed visual feedback in the mediolateral and anteroposterior directions affected postural control in the early post-stroke phase. In this pilot randomized controlled trial, 27 hemiparetic stroke patients were randomized to experimental (*n* = 13) and control (*n* = 14) groups. Dynamic sitting exercise (30 times/day, 5 days/week) in the mediolateral and anteroposterior directions, with 500-ms-delayed (experimental group) or real-time (control group) visual feedback on a computer, was added to usual physical therapy. We evaluated the postural assessment scale for stroke (PASS), static and dynamic sitting balance tasks, the five-times sit-to-stand test, trunk impairment scale, functional ambulation category, and functional independence measure–motor items. In intention-to-treat analysis, the experimental group demonstrated a significant intervention effect on the PASS score (*p* < 0.05). The mean percentage of body weight on the moving side in the lateral sitting task and the number of successes in the five-times sit-to-stand test were significantly higher in the experimental group than those in the control group (*p* < 0.05). Thus, the proposed exercise improves postural control, dynamic sitting balance, and sit-to-stand ability in early post-stroke patients.

## 1. Introduction

The balance ability of stroke patients is altered by motor paresis, sensory deficits, and spatial cognitive dysfunctions [1,2]. Visual feedback using a force platform is one of the most widely incorporated external stimuli for improving balance ability in stroke patients and has been shown to be particularly effective for standing balance practice [3,4,5,6]. The patient can use the real-time center of gravity to recognize its position and trajectory, which allows the patient to take appropriate strategies to stabilize postural control [5]. As for sitting balance, in recent years, it has been reported that functional improvement of postural control can be obtained in stroke patients by integrating visual feedback into well-structured individualized physiotherapy [7]. Therefore, it may be effective to use visual feedback in conjunction with individualized sitting balance exercises, which considers the time of onset and balance characteristics in patients with stroke.

Sitting ability in the early and subacute stages of stroke onset has been reported to be related to functional balance ability [8] and is an important early predictor of long-term walking ability [9,10,11], activities of daily living (ADL) [12], and depression [13]. In particular, it has been shown that lateral sitting balance reflects subsequent functional changes in the early post-stroke phase [14], and the effects of dynamic lateral sitting practice have been demonstrated [15]. In addition, the sitting balance in stroke patients during the same period shows non-paretic side predominant loading [11] and postural tilt to the non-paretic side [16], and maximum weight shift in the sitting position to the lateral side is often restricted to the direction of the paretic side [8]. In contrast, some patients show a postural tilt to the paretic side and predominant loading to the paretic side [8,16,17], and their ability to shift weight to the non-paretic side may be limited [18,19]. Therefore, the dynamic sitting balance in the early post-stroke phase differs across cases and requires individualized intervention.

Furthermore, in the early post-stroke phase, the ability to shift weight forward in the sitting position has been shown to be the most influential factor in the ability to perform sit-to-stand movements [20]. It has also been reported that sitting forward-reaching exercises can improve sit-to-stand movement ability, as a carryover effect [21]. The ability to sit-to-stand transition is a skill that is frequently required in daily life [22]. The inability to perform this movement is associated with falls in stroke patients [23]. Moreover, this ability is necessary before starting to walk [24]. Thus, it is important to improve the ability to perform this movement. Therefore, in patients who can maintain a sitting posture in the early post-stroke phase, improvement in the lateral (paretic or non-paretic side) and anterior dynamic sitting balance is considered to promote outcomes, such as postural control and basic movement ability (including the sit-to-stand movement).

The internal model [25,26] has been proposed for human motor control. The forward model [27] in this internal model is a system that predicts the sensory information of a movement before the actual sensory feedback is available (efference copy), which allows for online correction [28]. If there is an error between the sensory feedback and the efference copy, the motor control system works to correct this error in a feedforward manner [29]. Delayed visual feedback is a technology that delays real-time visual feedback and has been shown to stabilize balance ability in humans [30]. Adapting the principles of the internal model to delayed visual feedback, these external stimuli may activate rapid correction via the forward model by setting an error (delay) between the predicted visual information (efference copy) and the actual visual information. Thus, delayed visual feedback may be effective when combined with dynamic balance exercises, especially when a large amount of active movement is encouraged. However, there are no reports on the use of delayed visual feedback in clinical practice.

Therefore, we hypothesized that using individualized dynamic sitting exercises with delayed visual feedback could improve postural control, sit-to-stand ability, and lateral and anterior dynamic sitting balance in the early post-stroke phase. The purpose of this study was to examine the effects of dynamic sitting exercise in two directions with delayed visual feedback in the early post-stroke phase.

## 2. Materials and Methods

### 2.1. Study Design

This was a pilot double-blind randomized controlled trial that was conducted according to the CONSORT checklist [31]. Participants were randomly assigned to the experimental or control group after baseline assessment by an allocator who was not involved in the study, using the envelope method with computer-generated randomization. The study coordinator informed the physical therapist in charge of the intervention in the experimental or control group after being informed of group allocation by the allocator. Participants and outcome assessors were blinded to the intervention.

This study was approved by the Research Ethics Committees of the International Medical Center of Saitama Medical University (20-011) and the Tokyo Metropolitan University (21017). This study was registered with the University Hospital Medical Information Network Clinical Trials Registry (UMIN-CTR number: UMIN000040902). Written informed consent was obtained from all participants prior to their participation in the study.

### 2.2. Participants

Participants were first-ever stroke (infarction or hemorrhage) patients with hemiparesis who underwent inpatient rehabilitation at a university hospital in Japan from June 2020 to August 2021. The selection criteria were as follows: stroke onset within the previous 30 days, age > 20 years, ability to perform tasks, ability to maintain a sitting position without hand support for over 10 min, ability to understand instructions, no visual or visuospatial perception diseases, no history of neurological diseases other than stroke, and no history of orthopedic diseases that would interfere with assessment and intervention. Thus, those displaying severe impairment of consciousness or aphasia were not included.

### 2.3. Apparatus

**Figure 1:**
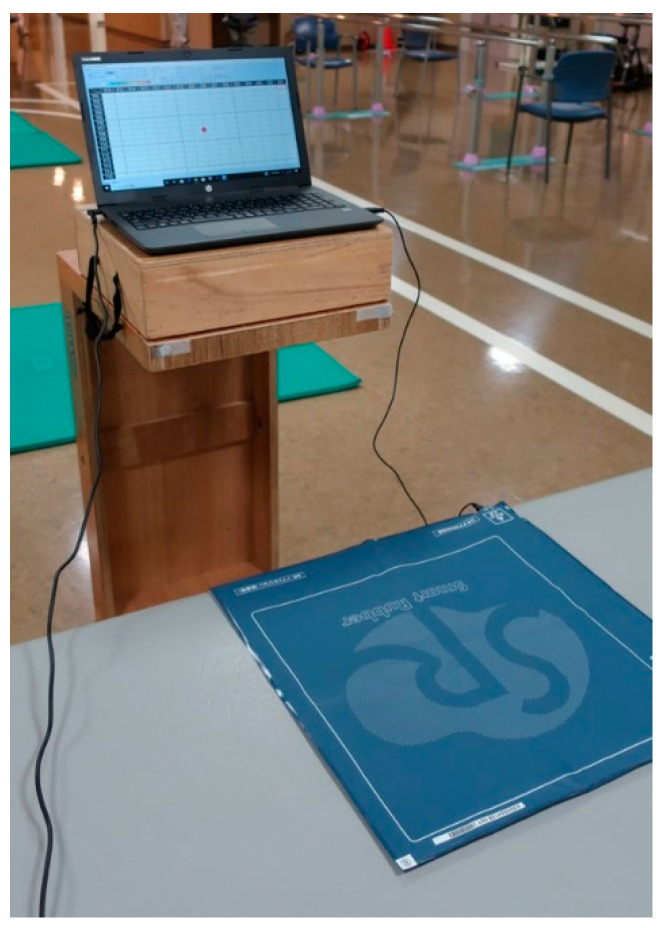
The Smart Rubber Soft Vision is a sheet-shaped apparatus that can be connected to a computer to perform posturographic examination and intervention using the center of pressure.

The Smart Rubber (SR) Soft Vision (Sumitomo Riko, Nagoya, Japan) was used to conduct intervention and evaluate outcomes (Figure 1). The SR Soft Vision is a sheet-shaped apparatus that conducts posturography and consists of 256 measurement points over a pressure-sensitive area of 350 mm × 350 mm with a sampling rate of 20 Hz. It can also be used in visual feedback regarding the center of pressure (COP) for dynamic sitting exercises, and the COP delay can be set every 1 ms. The output destination is on a computer screen. A 15.6-inch (approximately 37 cm × 26 cm) laptop computer (Hewlett-Packard, Palo Alto, CA, USA) was used in this study.

### 2.4. Intervention

The ability to shift weight to the lateral side (paretic or non-paretic) is restricted in the early post-stroke phase and varies among patients [8,18,19], and it is necessary to determine the laterality of the restriction when considering individualized physiotherapy. Therefore, a lateral sitting task [32] was performed prior to the intervention to determine the direction of movement for the lateral dynamic sitting exercises. In the lateral sitting task, the participants were asked to shift weight maximally from a static sitting position, without plantar contact, in the direction of the non-paretic or paretic side on a platform with SR Soft Vision and were instructed to hold the posture after shifting their weight. The time set for holding the posture after the maximum weight shift was 10 s, and the task was performed twice in each direction for the paretic and non-paretic sides.

**Figure 2:**
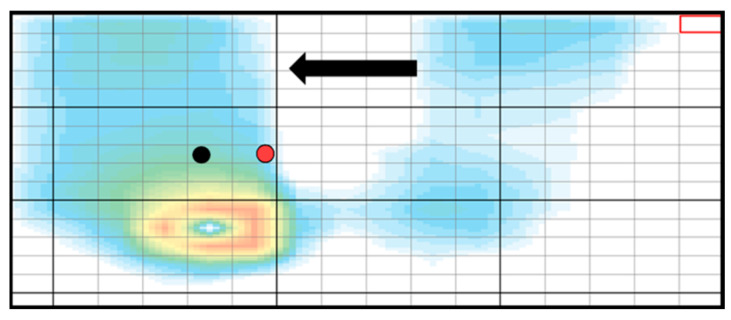
Center of pressure (COP) when moving in the direction of the arrow. The black circle indicates the actual COP and the red circle indicates the apparent COP (delayed COP).

The initial position in the intervention was a sitting posture with arms folded on a platform with SR Soft Vision, without plantar contact. The participants performed dynamic sitting exercises to the left, right, and forward while viewing the COP projected on a computer screen in front of them (Figure 2). For the experimental group, the visual feedback of the COP was delayed by 500 ms, while it was provided in real-time for the control group. Earlier studies have shown that humans are unable to notice the degree of feedback delay up to 600 ms [30]. Therefore, the degree of delay for the experimental group was set to 500 ms to ensure blinding of participants.

**Figure 3:**
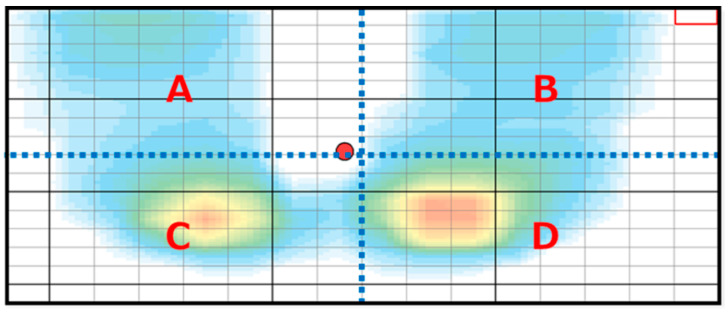
Computer screen output from the SR Soft Vision platform (Sumito Riko, Nagoya, Japan). The mean percentage body weight (BW) on the paretic side in the static sitting task, the mean percentage BW of the moving side in the lateral sitting task, and the mean percentage BW of the anterior direction in the forward sitting task were calculated from the amount of load in each of the A–D regions.

In lateral dynamic sitting exercises, the movement direction was to the side (paretic or non-paretic) which demonstrated a smaller mean load ratio in the lateral sitting task (Figure 3). The participants were instructed to shift their weight from the starting position to the maximum extent possible, without raising the hips on the non-moving side, and then to return to the starting position. In the forward dynamic sitting exercise, the participants were instructed to perform maximum trunk flexion, without falling forward, and return to the starting position. In the forward dynamic sitting exercise, a computer screen was set up at 45° below the resting gaze to allow for constant visualization of the COP as the trunk tilted forward.

Thirty sets of sitting exercises were performed per day, 5 days per week, in each movement direction. The physical therapist monitored the participant to prevent falls, especially in case of wobbling or danger of falling, and immediately held the participant for protection. Combined therapy included the usual physiotherapy, comprising a range of motion exercises, muscle strengthening, sit-to-stand, and walking exercises for both experimental and control groups. Combined therapy was performed according to stroke rehabilitation guidelines [33]. In addition, occupational therapy and speech-language therapy were provided, as needed, in addition to the usual physical therapy.

### 2.5. Outcomes

The primary outcome was the postural assessment scale for stroke (PASS) [34], which is a clinical visual assessment tool comprising 12 items (five items for maintaining posture and seven items for changing posture). The PASS has been reported to have good inter-rater reliability in the early post-stroke phase [35].

To assess static sitting balance, participants were taught to hold a comfortable posture for 30 s, with eyes open, without plantar contact on a platform with SR Soft Vision. From the obtained data, the mean velocity of the COP (cm/s) and mean percentage body weight (BW) on the paretic side (%) were calculated (Figure 3).

For the dynamic sitting balance, the mediolateral direction was the lateral sitting task, as described in the intervention section. For the anterior direction, participants performed the trunk flexion from the starting position to the front without falling over and were asked to hold the posture for 10 s after shifting their weight (forward sitting task) [32]. The mean velocity of the COP (cm/s) was also calculated in all dynamic directions (paretic and non-paretic sides and anterior) for 10 s. In the lateral sitting task, the mean percentage BW of the moving side (%) was calculated while holding the posture for 10 s, after shifting the weight to the paretic and non-paretic sides. In the forward sitting task, the mean percentage BW of the anterior direction (%: thigh) during the trunk flexion weight shift was calculated. The mean percentage BW on the paretic side in the static sitting task and that of the moving side in the lateral sitting task were calculated from the ratio of the total load in the A and C (left side) or B and D (right side) regions to the total load in the A to D regions (Figure 3). In addition, the mean percentage BW of the anterior direction in the forward sitting task was calculated as the ratio of the sum of the loads in the A and B regions to the sum of the loads in the A to D regions (Figure 3). The static and dynamic sitting tasks were performed twice each, and the mean of the two was adopted for analysis.

For the secondary outcomes, clinical measurements included the five-times sit-to-stand movement task [36], stroke impairment assessment scale (SIAS) [37], trunk impairment scale (TIS) [38], functional ambulation category (FAC) [39], and functional independence measure-motor (FIM-motor) items [40].

Generally, the five-times sit-to-stand movement task is measured by the time taken to perform the sit-to-stand movements [36]. However, as some early post-stroke phase patients in this study had difficulty performing these tasks [20], we considered the number of successes achieved in a five-times sit-to-stand movement task. Successful sit-to-stand movement was defined as a score of 3 in the sitting to standing up of the PASS sub-item.

The SIAS is a functional assessment of acute stroke patients [37] who are able to sit in a chair and was therefore considered suitable for the functional assessment of participants in this study. The SIAS assesses motor paresis, sensory deficits, trunk function, and unilateral spatial neglect, with a total score ranging from 0 to 76, with higher scores indicating better function.

The TIS is a scale used to assess trunk function in stroke patients [38]. It comprises three major items: static sitting balance (0–7 points), dynamic sitting balance (0–10 points), and coordination (0–6 points), with a total score ranging from 0 to 23 points, with higher scores indicating better trunk function.

The FAC is a visually assessable scale of gait ability [39], scored from 0 to 5 points, with higher scores indicating better gait ability.

The FIM-motor is a scale used to assess motor-related ADL [40]. It has a total of 13 items, each scored on a scale of 1–7 points, for a total score of 13–91 points. The higher the score, the better the ADL ability.

Feasibility was assessed by the attrition rate, adverse events associated with delayed visual feedback, and accuracy of blinding.

### 2.6. Statistical Analysis

SPSS version 26 (IBM; Armonk, NY, USA) was used for statistical analysis. For the intervention effect, the data, including missing data for the intention-to-treat (ITT) analysis, were analyzed using a linear mixed-effects model with random intercepts and slopes [41]. We included group, time, and their interaction as fixed effects and participants as random effects. If we found an interaction in the linear mixed model, we performed univariate comparisons between pre-intervention and post-intervention within each group. The Satterthwaite approximation was used to define the degrees of freedom. The significance level was set at 5%.

The sample size was based on previous research for the primary outcome [35] and was calculated using G*Power 3.1 (Heinrich Heine University, Dusseldorf, Germany) [42]. According to a significance level of 5%, an effect size of 0.89 and a power of 0.8 were set [35]; the required sample size was 42 participants. In addition, we estimated the attrition rate to be 15%, based on a previous study on sitting practice conducted at our hospital [43]; thus, we added 6 more participants for a target sample size of 48 participants. However, this study did not meet the required sample size due to the coronavirus disease (COVID-19) pandemic at our facility and was a pilot study. Therefore, a post hoc test in the ITT population was performed using G*Power 3.1 to determine the effect size and power (1−β) [42]. The effect size of the mean difference between the groups before and after the intervention was estimated using Cohen’s d. [44] The power of the test was calculated using the effect size and alpha error (0.05).

## 3. Results

**Figure 4:**
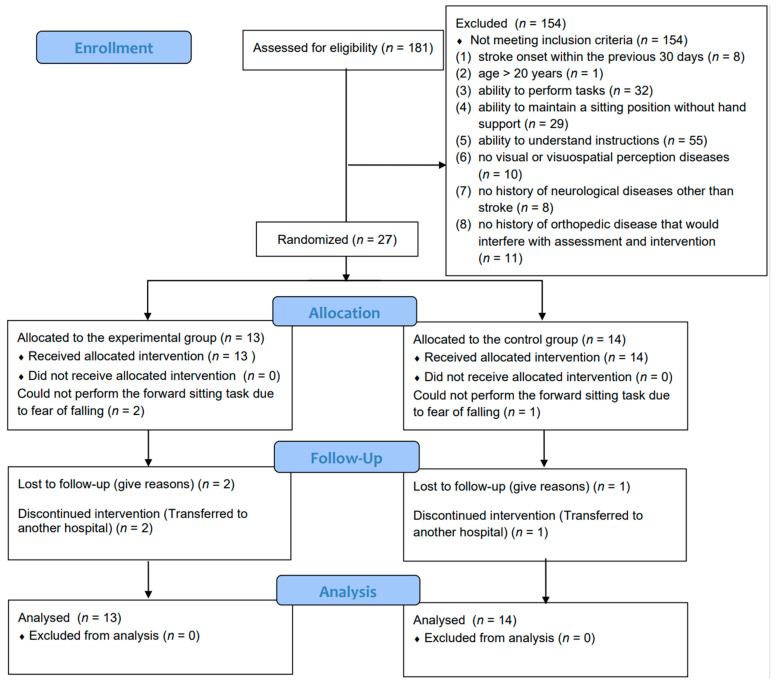
Flow diagram used for the selection of participants for this study.

Figure 4 shows the flow diagram of the trial participants. We screened 181 patients for eligibility between July 2020 and August 2021, of whom 27 met the inclusion criteria. Twenty-seven participants were randomly assigned to the experimental group (*n* = 13) or control group (*n* = 14). Three participants (two in the experimental group and one in the control group) could not perform the forward sitting task because of their fear of falling. Follow-up data were missing for three participants (one in the experimental group and two in the control group), all of whom withdrew because of discharge to another hospital during the study period. Therefore, the attrition rate was 15%. Participants’ demographic characteristics and baseline data are presented in Table 1. There were no significant differences in demographic characteristics between the two groups. No adverse events occurred during the study period. In addition, none of the participants were aware of the presence of a delay (ensuring blinding).

### Effects of the Intervention

In the main outcome, a significant interaction was observed in the PASS (F = 5.425, *p* = 0.030) (Table 2). The PASS score increased from 21.8 to 28.4 points in the experimental group and from 21.7 to 25.3 points in the control group. The univariate test showed significant improvements in both experimental and control groups (*p* < 0.001).

In the secondary outcomes of posturographic examinations, significant interactions were observed in the mean percentage BW on the paretic side in the lateral sitting task toward the paretic side (F = 5.832; *p* = 0.024) and that on the non-paretic side in the lateral sitting task toward the non-paretic side (F = 5.025, *p* = 0.035) (Table 2). The mean percentage BW on the paretic side in the lateral sitting task toward the paretic side increased from 69.7% to 84.3% in the experimental group and from 77.5% to 82.3% in the control group. In the univariate analysis, the experimental group showed a significant improvement (*p* < 0.001), while the control group did not (*p* = 0.176). The mean percentage BW on the non-paretic side in the lateral sitting task to the non-paretic side increased from 74.0% to 83.0% in the experimental group and from 76.8% to 77.7% in the control group. In the univariate analysis, the experimental group showed a significant improvement (*p* = 0.001), while the control group did not (*p* = 0.759).

In the clinical measurement of secondary outcomes, the number of successes in the five-times sit-to-stand test was significantly higher in the experimental group than in the control group (F = 5.728, *p* = 0.026) (Table 2). The number of successes in the five-times sit-to-stand test increased from 1.8 to 4.1 in the experimental group and from 2.1 to 2.9 in the control group. In the univariate analysis, the intervention group showed a significant improvement (*p* = 0.001), while the control group did not (*p* = 0.128). No significant interaction effects were observed for the other secondary outcomes (Table 2).

The post hoc calculations for the outcomes in the ITT population are shown in Table 3. Effect sizes ranged from 0.74 to 1.58, and power ranged from 0.46 to 0.98 in significant interaction outcomes.

## 4. Discussion

No previous randomized controlled trial has examined the effects of dynamic sitting exercises in two directions using delayed visual feedback in patients with stroke. The results of this study showed that, as hypothesized, the experimental group (using delayed visual feedback) developed better postural control and improved sit-to-stand performance, as a carryover effect, and improved lateral dynamic sitting balance than did the control group (using real-time visual feedback).

Patients in the early post-stroke phase have impaired sitting balance [1,8,14,16,17,18,19]. It has been reported that it is important to individualize sitting exercise when using visual feedback in combination with sitting exercise [7], and in this study, we assessed the characteristics of individuals’ sitting balance to develop an intervention strategy to enhance its effectiveness. The intervention used in this study can be adapted to many institutions and stroke patients because it can be implemented using a computer and a treatment table for hemiplegic early post-stroke patients who can maintain a sitting position.

The attrition rate in this study was 15%. The median length of stay in acute-care hospitals for stroke patients in Japan is approximately 20 days [45]. The length of stay tends to be slightly longer for patients who are actively rehabilitated. Because they were transferred to another hospital, the length of stay was generally 30 days for the participants in this study. In acute-care hospitals in Japan, as patients may be transferred in the middle of the intervention period, the results of shorter-duration interventions, as performed in this study, need to be demonstrated. In the present study, three participants were transferred to another hospital in the middle of the study period. However, the attrition rate was less than that of other intervention studies (15% to 33%) [15,43,46] conducted at the same institution in the past. Thus, the length of the current intervention did not contribute to the attrition rate. On the other hand, three participants had difficulty performing the forward sitting task due to fear of falling. No adverse events related to delayed visual feedback, such as mood discomfort or falls, were observed, and safety was ensured. In addition, none of the participants noticed whether the COP feedback was delayed or given in real-time, demonstrating that blinding was ensured. Considering these factors, the feasibility of all but the outcome of the dynamic sitting task in the anterior direction was considered to be high.

In this study, the experimental group using delayed visual feedback showed an improved ability to shift lateral bodyweight to the paretic and non-paretic sides in the posturographic examination, compared with that in the control group, using real-time visual feedback. However, there was no significant difference in the degree of improvement in the ability to shift weight forward between the two groups. Lakhani et al. reported that balance exercise with visual feedback for humans was effective but did not produce a carryover effect in fundamental research [47]. In the present study, the group that used real-time visual feedback also had a small carryover effect with respect to the lateral sitting task. In contrast, the group that used delayed visual feedback showed a carryover effect. The use of delayed visual feedback is based on feedback error learning [29] using an internal model. The experimental group required large trunk movements in the sitting posture to compensate for the error between the predicted visual information and delayed moving visual information. This dynamic trunk movement may have resulted in improved lateral sitting balance as a carryover effect. On the other hand, the loading of the paretic side in the lateral sitting task may have been affected by the difference in values at baseline, in addition to the intervention effect, which needs to be resolved in future studies with more participants. The lack of difference in the ability to shift the weight forward was thought to be due to the small distance traveled by the COP in the vertical direction, because of the horizontal shape of the display, and because the direction of restriction (front or back) was not assessed, unlike for the lateral direction.

We found that the dynamic sitting exercise with delayed visual feedback improved the PASS score compared to that for such exercise with real-time visual feedback. The minimum detectable change (MDC) in the PASS score in stroke patients in a study contemporaneous with the present study was reported to be 2.2 points [48]. The change in the PASS score before and after the intervention in this study was 6.6 points in the experimental group and 3.6 points in the control group. Both groups outperformed the MDC; however, the extent of the difference was greater in the experimental group. Sitting balance in the mediolateral direction has been shown to be associated with trunk function and standing balance [8,14], and sitting exercise in the mediolateral direction has been reported to improve trunk function [15]. Therefore, dynamic sitting balance in the mediolateral direction was improved by dynamic sitting exercise using delayed visual feedback, which improved PASS, an overall assessment of trunk function, and balance was thought to have improved in the experimental group.

In this study, the ability to perform sit-to-stand movements improved significantly in the group that received delayed visual feedback. In a previous study, the ability to shift weight forward in the sitting position had the greatest impact on the ability to perform sit-to-stand movements in early post-stroke patients [20]. However, in the present study, the forward sitting task did not differ between the two groups, and both experimental and control groups improved significantly. In contrast, the sitting balance to the lateral side improved significantly in the delayed visual feedback group only. Therefore, stabilization of lateral sitting balance, in addition to the ability to shift weight forward, may be important for sit-to-stand movements in the early post-stroke phase. Furthermore, sit-to-stand movements cannot be performed successfully unless the standing posture is stable. In patients with severe motor paresis and limited participation of the paretic lower limb in postural control, the non-paretic side is important for postural control in the standing posture [49]. Based on these findings, both the ability to shift weight forward and stable sitting balance to the lateral side may affect the sit-to-stand movements in the experimental group.

This study has several limitations. First, interactions were obtained for several outcomes, but the effect size was small. In addition, differences in some outcomes at baseline were observed. Second, three participants were unable to perform the forward sitting task due to fear of falling. Third, in this study, the sit-to-stand ability was evaluated based on the number of successes rather than the time taken to perform the task. Moreover, qualitative evaluations, such as the degree of loading on the paretic or non-paretic side and the degree of forward weight transfer during sit-to-stand movements, were not conducted. Fourth, there was no follow-up evaluation. The median length of stay in acute-care hospitals in Japan is 20 days [45], which makes it difficult to conduct follow-up evaluations. Lastly, the number of cases did not reach the target sample size. The COVID-19 pandemic made it difficult for us to continue the study. Therefore, this is a pilot study, and we need to conduct another study with a larger number of cases in the future.

## 5. Conclusions

These results suggest that dynamic sitting exercise in both mediolateral and anteroposterior directions with delayed visual feedback could improve postural control, dynamic sitting balance, and sit-to-stand ability in participants in the early post-stroke phase, providing insight into improved post-stroke rehabilitation approaches. Feasibility was also noted. Nevertheless, it is necessary to verify the effectiveness of this intervention in large-scale multicenter studies.

## Figures and Tables

**Table 1:** Demographic characteristics of the participants at baseline.

	Experimental Group	Control Group	*p* Value
Sex (female/male)	4/9	4/10	0.901
Age (years), mean ± SD	67.2 ± 14.3	66.1 ± 11.6	0.841
Lesion side (right/left)	6/7	9/5	0.343
Etiology (infarction/hemorrhage)	8/5	7/7	0.547
Days from stroke onset (days), mean ± SD	14.2 ± 4.0	17.5 ± 6.9	0.148
Length of hospital stay (days), mean ± SD	29.6 ± 7.4	31.9 ± 10.2	0.508
SIAS-motor (0/1/2/3/4/5)			
Upper limb			
Knee to mouth	5/1/1/4/2/0	3/3/3/2/3/0	
Finger function	6/1/0/4/2/0	5/3/2/1/3/0	
Lower limb			
Hip flexion	4/3/0/4/2/0	1/4/3/3/3/0	
Knee extension	4/2/2/2/3/0	5/1/0/4/4/0	
Foot tap	8/0/0/2/3/0	5/1/3/4/1/0	
Movement direction of lateral dynamic sitting exercise (paretic side/non-paretic side)	8/5	7/7	0.547

Abbreviations: SD, standard deviation; SIAS, stroke impairment assessment set.

**Table 2:** Differences in outcomes between the two groups.

	Experimental Group		Control Group		Group Effect		Time Effect		Interaction Effect	
Outcomes	Pre-Intervention	Post-Intervention	Pre-Intervention	Post-Intervention	F Value (df)	*p* Value	F Value (df)	*p* Value	F Value (df)	*p* Value
Main outcome										
PASS (Primary outcome)	21.8 (18.3, 25.2)	28.4 (24.9, 31.9)	21.7 (18.4, 25.0)	25.3 (21.9, 28.8)	0.467 (1, 25)	0.501	63.299 (1, 21)	<0.001 *	5.425 (1, 21)	0.030
Secondary outcome										
Static sitting task										
Mean velocity of COP (mm/s)	1.7 (1.3, 2.2)	1.3 (0.8, 1.8)	2.1 (1.6, 2.5)	1.5 (1.0, 2.0)	0.756 (1, 26)	0.392	9.039 (1, 24)	0.006	0.202 (1, 24)	0.657
Mean percentage BW on the paretic side (%)	50.4 (46.4, 54.4)	47.7 (43.5, 51.8)	46.9 (43.1, 50.7)	50.0 (45.8, 54.3)	0.071 (1, 23)	0.793	0.011 (1, 22)	0.917	2.536 (1, 22)	0.125
Lateral sitting task to the paretic side										
Mean velocity of COP (mm/s)	2.8 (1.8, 3.7)	2.8 (1.8, 3.7)	3.6 (2.6, 4.5)	3.4 (2.4, 4.3)	1.296 (1, 25)	0.266	0.235 (1, 21)	0.633	0.251 (1, 21)	0.622
Mean percentage BW on the paretic side (%)	69.7 (64.0, 75.3)	84.3 (78.5, 90.2)	77.5 (72.1, 83.0)	82.3 (76.4, 88.3)	0.719 (1, 26)	0.404	22.540 (1, 24)	<0.001 *	5.832 (1, 24)	0.024
Lateral sitting task to the non-paretic side										
Mean velocity of COP (mm/s)	2.4 (1.9, 2.9)	2.4 (1.9, 2.9)	2.6 (2.2, 3.1)	2.2 (1.7, 2.8)	0.014 (1, 25)	0.907	1.104 (1, 23)	0.304	0.817 (1, 23)	0.375
Mean percentage BW on the non-paretic side (%)	74.0 (68.8, 79.2)	83.0 (77.7, 88.4)	76.8 (71.8, 81.8)	77.7 (72.3, 83.2)	0.157 (1, 24)	0.696	7.629 (1, 22)	0.011	5.025 (1, 22)	0.035
Forward sitting task										
Mean velocity of COP (mm/s)	2.6 (1.7, 3.5)	2.6 (1.7, 3.5)	3.3 (2.4, 4.1)	3.5 (2.6, 4.4)	1.910 (1, 22)	0.181	0.179 (1, 19)	0.677	0.270 (1, 19)	0.609
Mean percentage BW on the anterior direction (%)	38.3 (34.4, 42.2)	54.8 (50.8, 58.8)	36.6 (33.0, 40.2)	59.3 (55.4, 63.3)	1.199 (1, 19)	0.287	70.087 (1, 22)	<0.001 *	1.757 (1, 22)	0.199
Five-times sit-to-stand task	1.8 (0.7, 3.0)	4.1 (3.0, 5.3)	2.1 (1.1, 3.2)	2.9 (1.7, 4.0)	0.464 (1, 25)	0.502	21.566 (1, 22)	<0.001 *	5.728 (1, 22)	0.026
SIAS	49.2 (42.9, 55.4)	52.0 (45.7, 58.3)	48.9 (42.8, 54.9)	52 (45.5, 57.7)	0.007 (1, 25)	0.934	27.722 (1, 21)	<0.001 *	0.011 (1, 21)	0.917
TIS	12.1 (9.6, 14.6)	17.2 (14.6, 19.7)	13.0 (10.6, 15.4)	16.0 (13.5, 18.6)	0.005 (1, 25)	0.945	40.875 (1, 22)	<0.001 *	2.676 (1, 22)	0.116
FAC	1.8 (1.4, 2.3)	2.4 (2.0, 2.9)	1.8 (1.4, 2.2)	2.2 (1.8, 2.7)	0.175 (1, 26)	0.680	23.910 (1, 22)	<0.001 *	0.312 (1, 22)	0.582
FIM-motor	39.2 (32.2, 46.2)	45.9 (38.8, 52.9)	37.6 (30.9, 44.4)	46.7 (39.8, 53.6)	0.006 (1, 25)	0.938	50.5 (1, 21)	<0.001 *	1.223 (1, 21)	0.281

Continuous data are expressed as mean (95% confidence interval) of the estimate. Abbreviations: BW, body weight; COP, center of pressure; PASS, postural assessment scale for stroke; SIAS, stroke impairment assessment Set; TIS, trunk impairment scale; FAC, functional ambulation category; FIM-motor, functional independence measure-motor. * Statistically significant difference (*p* < 0.05).

**Table 3:** Post hoc power calculations for outcomes in the ITT population.

Outcomes	Power (1−β)	Effect Size	Mean Differences
Primary outcome			
PASS	0.55	0.84	3.08
Secondary outcome			
Static sitting task			
Mean velocity of COP (mm/s)	0.07	0.18	0.15
Mean percentage BW on the paretic side (%)	0.05	0.04	0.41
Dynamic sitting task to the paretic side			
Mean velocity of COP (mm/s)	0.06	0.09	0.11
Mean percentage BW on the paretic side (%)	0.46	0.74	9.95
Dynamic sitting task to the non-paretic side			
Mean velocity of COP (mm/s)	0.13	0.33	0.36
Mean percentage BW on the non-paretic side (%)	0.98	1.58	8.07
Dynamic sitting to the anterior direction			
Mean velocity of COP (mm/s)	0.28	0.56	0.21
Mean percentage BW on the anterior direction (%)	0.19	0.43	6.56
Number of successes in the five-times sit-to-stand task	0.51	0.80	1.62
SIAS	0.05	0.03	0.11
TIS	0.35	0.63	2.19
FAC	0.07	0.15	0.12
FIM-motor	0.13	0.34	2.50

Abbreviations: ITT, intention-to-treat; BW, body weight; COP, center of pressure; PASS, postural assessment scale for stroke; SIAS, stroke impairment assessment set; TIS, trunk impairment scale; FAC, functional ambulation category; FIM-motor, functional independence measure-motor.

## Data Availability

The datasets used and/or analyzed during the present study are available from the corresponding author on reasonable request.
